# Robust Intra-Body Communication Using SHA1-CRC Inversion-Based Protection and Error Correction for Securing Electronic Authentication

**DOI:** 10.3390/s20216056

**Published:** 2020-10-24

**Authors:** Seongho Cho, Daejin Park

**Affiliations:** 1 School of Electronic and Electrical Engineering, Kyungpook National University, Daegu 41566, Korea; rpoets@lgdisplay.com; 2 LG Display Co., Ltd., Gumi 39394, Korea; 3 School of Electronics Engineering, Kyungpook National University, Daegu 41566, Korea

**Keywords:** intra-body communication, electrostatic coupling, error correction, securing electronic authentication, secure hash algorithm 1

## Abstract

The explosive increase in the number of IoT devices requires various types of communication methods. This paper presents secure personal authentication using electrostatic coupling Intra-body communication (IBC) based on frequency shift keying (FSK) and error correction. The proposed architecture uses GPIO for a transmitter and analog-to-digital conversion (ADC) for a receiver. We mplemented FSK modulation, demodulation, data protection, and error correction techniques in the MCU software without applying hardware devices. We used the characteristic that the carrier signal is 50% duty square wave for 1-bit error correction and applied a method of randomly inverting SHA1 hash data to protect user authentication data during transmission. The transmitter modulates binary data using a square wave as a carrier signal and transmits data through the human body. The receiver demodulates the signal using ADC and decrypts the demodulated binary data. To determine the carrier frequency from ADC results, we applied a zero-crossing algorithm which is used to detect edge characteristics in image processing. When calculating the threshold value within the zero-crossing algorithm, we implemented an adaptive threshold setting technique utilizing Otsu’s binarization technique. We found that the size of the electrode pad does not affect the signal strength, but the distance between the electrode pad and the skin has a significant effect on the signal strength. Our results show that binary data modulated with a square wave can be successfully transmitted through the human body, and, when 1-bit error correction is applied, the byte error rate on the receiver side is improved around 3.5% compared to not applying it.

## 1. Introduction

Today, people use a variety of digital devices, such as smartphones, smartwatches, wearable, and healthcare devices. They can exchange digital information between devices anytime, anywhere via Wi-Fi or mobile networks. In the world of healthcare, there is currently a large demand for remote and continuous patient monitoring [1,2,3,4]. The Internet of Things (IoT) is a network of devices, sensors, and other items of various functionalities that interact and exchange data electronically. The explosive growth in the number of IoT devices (estimated to be 50 billion by 2020) requires various types of communication methods [5,6,7,8,9,10,11].

Zimmerman introduced a system to send and receive digital information through the human body in 1996 without using RF-based communication networks such as Wi-Fi, mobile communication, and Bluetooth or using a wired cable [12]. He proposed a wireless communication system that allowed electronic devices on and near the human body to exchange digital data through near field electrostatic coupling. Digital information was transmitted by modulating electric fields and electrostatic coupling currents through the human body. Intra-Body Communication (IBC) has advantages in terms of security, outside radio interference, and energy efficiency because the communication area is limited to the human body compared to other wireless communication. Based on this potential, research on modeling and high-frequency characteristics of the human body as a communication channel has been actively conducted [13,14,15,16,17].

Nippon Telegraph and Telephone Corporation (NTT), introduced RedTacton technology supporting IEEE 802.3 half-duplex in 2005. It achieved a communication speed of 10 Mbps by implementing a transceiver with an electromagnetic field sensor composed of an electro-optic crystal and a laser [18]. However, this method requires high cost and high power consumption and requires an additional pad for ground connection in addition to an electrode pad for transmission and reception. In another study, a wideband signaling transceiver chip was fabricated to improve communication speed and low-power consumption. It achieved a 2 Mbps communication speed using an electrostatically coupled IBC method [19]. It recovers clock and data information from a received signal that is distorted by the human body and has a low signal strength by employing a clock and data recovery (CDR) circuit. A 10.7 MHz FM transmission IC was applied to implement a transceiver that communicated heart rate and oxygen saturation data from a measurement module [20].

While many hardware-based systems exist to implement IBC [18,19,20], this paper demonstrates the use of software implementation for IBC system. The only hardware used is a general MCU, GPIO for a transmitter and ADC for a receiver. The FSK modulation, demodulation, and error correction operations are implemented entirely in the MCU software. We focus on a software-based electrostatic coupling IBC system for personal authentication based on FSK. Additionally, to enhance security, we propose a 1-bit error correction algorithm and random inversion-based SHA1 with CRC code.

We presented our initial work regarding software-based IBC feasibility previously [21]. We expanded on the initial version by adding personal identification data protection and adaptive threshold techniques with further experimental results. The major contributions of this paper are as follows: (1) introducing and exploring the concept of electrostatic coupling IBC; (2) showing how to implement FSK modulation and random data inversion-based SHA1 with CRC code, as well as how how carrier signals can be transmitted through the human body; (3) showing how to implement FSK demodulation and 1-bit error correction; and (4) designing and implementing a transmitter and receiver software for the IBC system.

## 2. Background

In IBC technology, the human body acts as a special kind of transmission channel. The basic theory of IBC is that a weak electric field is induced into the human body to transmit a signal between devices that are in the impedance of Wegmueller et al. [22] or in direct contact with the human body. Two conceptually different approaches to motivate the electrical signal onto the human body have been proposed: (1) electrostatic coupling; and (2) a galvanic coupling.

In electrostatic coupling, energy flows from a transmitter to a receiver when they share the same electric field. This electric field of the human body causes an induced current flow from a transmitter to a receiver through electrodes. An electrode that transmits and receives signals is attached to the human body, and the transmission signal is electrostatically coupled through the human body to reach the receiver. The human body acts as a conductor and the floating ground of transmitter and receiver acts as a return path for the signal. In this method, signal and ground are coupled to the environment, so the signal can be distorted by environmental conditions.

In Zimmerman’s research, a narrowband transmission method such as on–off keying was applied to achieve a communication speed of 2.4 kbps, and a method of recovery through retransmission when an error occurred during communication was adopted. Kurt Partridge et al. [23] designed and implemented an IBC system modeled after Zimmerman’s original design and extended it to measure signal strength. He used quantitative measurements of data error rates and signal strength while varying distance, electrode location on the human body, plate size and shape, and several other factors. In his work, plate size and shape have only minor effects, but the distance to the plate and the coupling mechanism significantly affect signal strength. The transceiver size was 8 cm × 13 cm, and he achieved a 56 kbps data rate.

In galvanic coupling, the differential signal created by a pair of electrodes is transmitted through the human body and the signals are captured by another pair of electrodes at the receiver. There are two electrodes on both the transmitter and receiver sides. A pair of transmitting electrodes generates an alternating current, which is injected into the skin and acts like a wire. This alternating current generates a voltage across a pair of receiving electrodes on the human body. The difference from electrostatic coupling is that alternating current is coupled inside the human body instead of between the human body and the environment. Because the signal is completely contained within the human body and it is not required external ground reference, its performance is not affected by the surrounding environment [24]. However, it has been shown that it works with short distances (under 15 cm) between the transmitter and the receiver [25].

IBC coupling methods are low frequency (under 50 MHz) and low power compared to conventional high-frequency electromagnetic signals that go up to several GHz [26]. For this reason, coupling methods comply with safety, reduce energy consumption, and prevent body antenna effect. For safety reasons, the injected current must be low enough not to damage tissues and nerves, especially when applied for long periods [27].

## 3. Proposed Architecture

**Figure 1:**
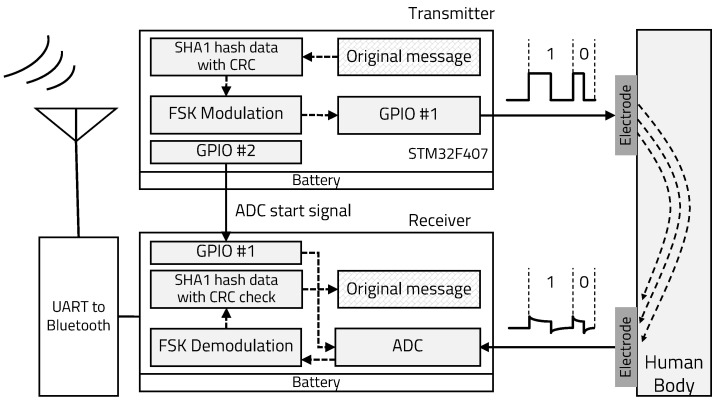
Electrostatic coupling IBC system structure.

This section describes the personal identification data protection technique, the FSK modulation and demodulation method, and 1-bit error correction for the IBC system. In our proposed IBC system based on FSK, the transmitter encrypts original message data based on SHA1 containing the CRC code and transmits encrypted binary data using a 50% duty square wave as a carrier signal through the human body. The receiver demodulates the carrier signal by checking its frequency and decrypts the binary data. To effectively extract the edge timing information of the square wave carrier signal, a zero-crossing algorithm and the adaptive threshold technique are applied. We implemented a software-based IBC system by utilizing GPIO (transmitter) and ADC (receiver) built in a general purpose MCU. One trigger signal is connected between transmitter and receiver for synchronization because of software limitation and this signal is transmitted to the receiver just before every 1-byte transmission, and the receiver starts ADC right after receiving the trigger signal. Figure 1 shows the overall structure of the proposed IBC system for personal authentication.

### 3.1. Personal Identification Data Protection Using Random Data Inversion-Based SHA1-CRC

Personal authentication through handheld or wearable devices is always exposed to attacks on information data. Various studies are being conducted to eliminate these security threats. To protect against attacks, efficient key agreement protocols over temporal confidential and authenticated channels and a transitive authentication system for multiple devices were proposed by Chen et al. [28]. In addition, an improved authentication protocol between multi-server distributed cloud and IoT devices through 5G network was proposed by Wu et al. [29]. Chien-Ming et al. [30,31] reported an improved anonymous mutual authentication key schemes that can protect against various types of attacks in wireless body area networks.

Original message data encryption is critical to applying an IBC system for securing personal authentication. SHA1 is a one-way function, so it is very difficult to decrypt the original message data by using an inversion function. Therefore, we adopted a lightweight SHA1 procedure to generate a 160-bit binary data sequence for original personal identification data on the transmitter. The receiver has a lookup table for the corresponding personal identification data, so it can verify who is requesting authentication. We applied a SHA1-CRC inversion-based data protection technique [32,33,34]: whether or not to invert binary data encrypted by SHA1 (except CRC value) is randomly determined, making it difficult to identify SHA1 generation patterns even if the same message is repeatedly transmitted. This method not only has a small overhead but also enhances security through a simple procedure.

**Figure 2:**
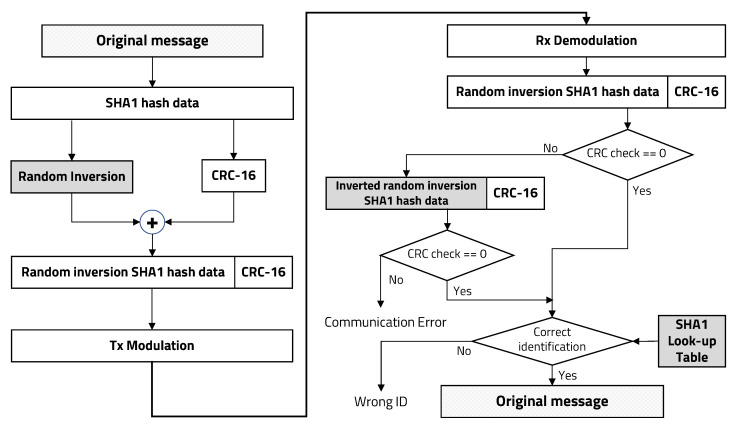
User identification data protection using random inversion-based SHA1-CRC.

To decrypt randomly inverted SHA1 hash data, the receiver performs CRC check from the two types of data received and the inverse received SHA1 hash data, and then it determines whether the received data is correct. The receiver divides the received binary data by CRC polynomial and verifies whether the remainder is zero. If it is not zero, the receiver divides the inverse received SHA1 hash data once more by the CRC polynomial. In both cases, if nonzero, the receiver determines that the received data are corrupted or a communication error has occurred. If the receiver determines that there are no errors during communication, the SHA1 hash data are compared to the SHA1 lookup table for personal authentication. Figure 2 shows the overall procedure of binary data protection using this SHA1-CRC inversion-based IBC system for personal secure electronic authentication.

### 3.2. Transmitter Design

The proposed IBC system directly transmits a digital signal through a transmitter into the human body, transfers a carrier signal over the human body channel, then demodulates the digital signal at the receiver. It enables the use of only a single electrode for data transmission. The strength of the received signal through the human body is not constant depending on the surrounding environment and noise conditions. Amplitude shift keying and on-off keying methods demodulate data based on a difference in the strength of a received signal and are easily influenced by signal attenuation. Thus, when the strength of the received signal changes due to external interference, demodulation is difficult. On the other hand, FSK is robust to transmission channel conditions because it contains information on carrier frequencies. There is no frequency change even if attenuation, distortion, or interference occur in the transmission line.

**Figure 3:**
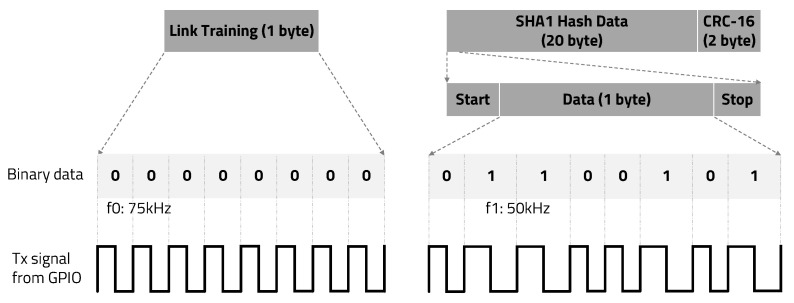
Packet structure and binary data FSK modulation.

The proposed FSK modulation and demodulation method for our IBC system is not sensitive to the strength or fluctuation of the received signal. Even if a 1-bit error occurs due to ambient noise, the error bit can be recovered by utilizing the characteristics of the carrier signal with a duty ratio of 50% and adjacent edge information. Because of these advantages, in this paper, the binary FSK technique is applied for an electrostatic coupling IBC system and the transmitter is implemented using GPIO of general purpose MCU. As shown in Figure 3, bit 1 was set to a carrier frequency of 50 kHz, and bit 0 was set to 75 kHz. The transmitter and receiver exploiting FSK are connected to the human body with a single aluminum metal electrode.

### 3.3. Receiver Design

#### 3.3.1. Carrier Frequency Extraction Using Edge Detection Algorithm

In the FSK communication, it is clear that the carrier frequency can be found through the wavelength. First, a receiver performs ADC on the received signal electrostatically coupled by the human body and performs software demodulation by detecting the period of the carrier signal from the ADC results. To detect the carrier frequency, it is critical to accurately detect the edge transition timing of the square wave.

**Figure 4:**
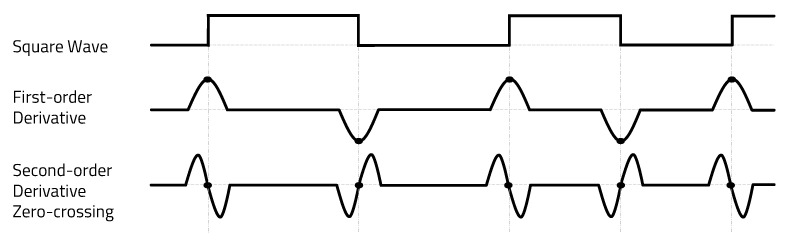
Zero-crossing detect.

The zero-crossing concept was applied to clearly extract the edge transition characteristics from the ADC results, as shown in Figure 4. One method of detecting zero-crossing in discrete signals is to compare the sign of the current sample with the sign of the previous sample. As zero-crossing detection only uses the points of the carrier signal where it crosses zero, it is insensitive to clipping and carrier frequency deviation, as long as the carrier frequency is constant. The time interval at which the carrier signal crosses zero is inversely proportional to the frequency. Therefore, it is possible to determine the carrier frequency by measuring the zero-crossing time interval, so the receiver can be implemented very simply.

Algorithm 1 represents the edge detection operation from ADC results. Depending on the ADC configuration of the receiver board, it is stored continuously at regular time intervals. Then, the second-order differential coefficients are calculated from the ADC results and stored in array *D*, and the stored values are sequentially searched. When there is a change in sign between successive second-order differential coefficients and the amount of change is more than a threshold, it is judged as an edge and stored in memory in the format below (in this paper, this is called “Edge information”).

Edgeinformation=(Index,Edgepolarity)

where: 

Index = Array *D*’s index judged as an edge

Edgepolarity = Positive edge is saved as 1, Negative edge is saved as −1
**Algorithm 1:** Edge detection algorithm.
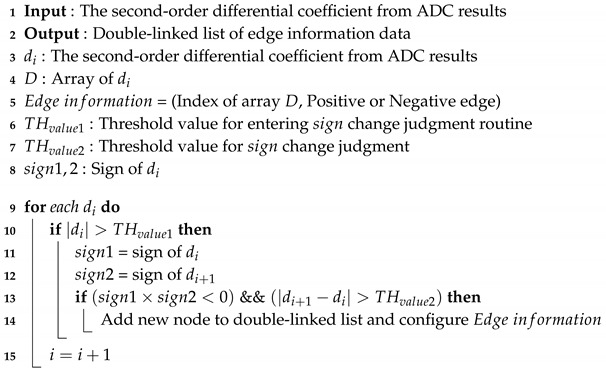


Edge information is stored by implementing a double-linked list that facilitates node addition and data search. Whether it is a positive or negative edge is used to recover edge information of error bits occurred during communication, and the index is used to determine the carrier frequency by calculating the index delta between consecutive positive edges. In general, when using the second-order derivate, noise is amplified, so we applied a two-stage high-pass filter for noise filtering in Algorithm 1. In the first stage, it is possible to enter the sign change judgment routine only when the second-order differential coefficient change amount is above the threshold. In the second stage, it can be judged as an edge only when it has both a rate of change above the threshold and the sign change, as shown in Algorithm 1.

#### 3.3.2. Adaptive Threshold Technique for Edge Detection Algorithm

The threshold value is a very critical parameter for the edge detection Algorithm 1. If the optimal threshold cannot be decided automatically, edge detection algorithms are rarely used in real applications. Therefore, the dynamic threshold decision is very important in our IBC system.

In image processing, binarization refers to the conversion of pixels that are brighter than a given threshold to all white, and all pixels below the threshold to be black, to distinguish objects from the background. In theory, the threshold that minimizes classification errors is called the optimal threshold. That is, when a certain value of T is fixed, T, which minimizes the sum of the proportion of the object pixels darker than T and the proportion of background pixels lighter than T, is called an optimal threshold. However, this only represents a theoretical goal, not a method for obtaining an actual threshold. This is because, to know whether T is optimal, it is necessary to know in advance which pixel is the object and which is the background. Otsu’s method is the most practical method to find the threshold value by looking at the brightness distribution of the actual input image [35,36]. Otsu’s binarization method finds a T value that minimizes within-class variance between two classes or maximizes between-class variance when image pixels are classified into two classes based on threshold T. Similarly, we can apply this binarization method for optimal threshold decision in the edge detection algorithm, which determines signals with an amount of change greater than a given threshold as edges (objects); otherwise, the signals are defined as the noise (background).

Let the absolute values of the second-order differential coefficients from ADC results be represented in M values [1, 2, ..., M]. The number of second-order absolute differential coefficients at value *i* is denoted by $z_{i}$ and the total number of the second-order differential coefficients can be expressed as $Z=z_{1}+z_{2}+\cdots +z_{M}$. These values are separated into two classes, $E_{0}$ and $E_{1}$ (e.g., noise and edge) by a threshold T. $E_{0}$ represents the value of [1, 2, ..., T] and $E_{1}$ represents the value of [T+1, T+2,..., M]. The probabilities of class occurrence and the class mean values can then be written as the equations below.

$$p\left(i\right)\;=\;z_{i}/Z,z_{i}>0,\;\;\omega_{0}\;=\;\sum_{i=1}^{T}p(i)\;=\;\omega \left(T\right),\;\;\omega_{1}\;=\;\sum_{i=T+1}^{M}p(i)\;=\;1-\omega \left(T\right)$$

where

$\omega_{0}$ = Probabilities of noise class

$\omega_{1}$ = Probabilities of edge class

$$\mu_{0}\;=\;\sum_{i=1}^{T}\frac{ip(i)}{\omega_{0}},\;\;\mu_{1}\;=\;\sum_{i=T+1}^{M}\frac{ip(i)}{\omega_{1}}$$

where

$\mu_{0}$ = Average of noise class

$\mu_{1}$ = Average of edge class

The class variances are given by as below equations.

$${\sigma_{0}}^{2}\;=\;\sum_{i=1}^{T}\frac{{\left(i-\mu_{0}\right)}^{2}p\left(i\right)}{\omega_{0}},\;\;{\sigma_{1}}^{2}\;=\;\sum_{i=T+1}^{M}\frac{{\left(i-\mu_{1}\right)}^{2}p\left(i\right)}{\omega_{1}}$$

where

${\sigma_{0}}^{2}$ = Variance of noise class

${\sigma_{1}}^{2}$ = Variance of edge class

Between-class variance and within-class variance are calculated as follows. Minimizing the value of within-class variation is the same as maximizing the value of between-class variation. Either criterion may be used, but, computationally, it is efficient to maximize the value of between-class variation.

(1) $${\sigma_{W}}^{2}\;=\;\omega_{0}{\sigma_{0}}^{2}+\omega_{1}{\sigma_{1}}^{2}$$

(2) $${\sigma_{B}}^{2}\;=\;\omega_{0}\omega_{1}{\left(\mu 1-\mu 2\right)}^{2}$$

where

${\sigma_{W}}^{2}$ = Within-class variance

${\sigma_{B}}^{2}$ = Between-class variance

To determine the optimal threshold value in the edge detection Algorithm 1, we applied a link training session before transmitting valid data, as shown in Figure 3. Link training is the first stepping stone to enabling the communication channel between transmitter and receiver. During a link training session, the transmitter sends 0 × 00 data (except start and stop bits) and the receiver calculates the second-order differential coefficients from ADC results and between-class variance. Determining $TH_{value1}$ in the edge detection algorithm requires the receiver to find the value at which between-class variance is maximized or within-class variance is minimized. Minimizing Equation (1) is the same as maximizing Equation (2). Either equation can be used, but it is computationally efficient to maximize Equation (2). It is possible to calculate this using Equation (2) from 1 to max absolute value of the second-order differential coefficients.

**Figure 5:**
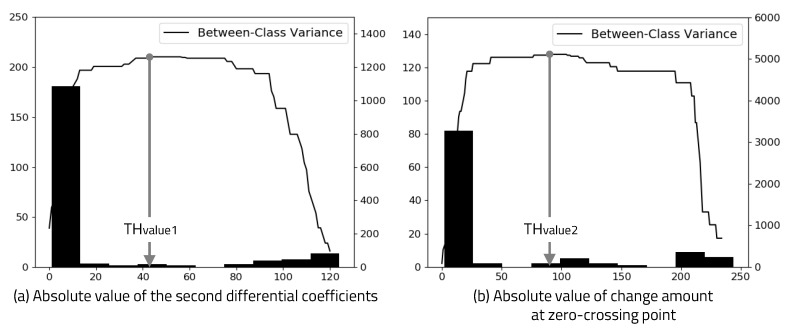
Histogram and between-class variance.

The 0 × 00 data for the link training session consist of a total of 18 edges. The value to maximize between-class variance can be obtained by calculating the 19th value ±5 of the second-order differential coefficients sorted in descending order. The value at which between-class variance is maximized is $TH_{value1}$, and this method is more efficient in terms of computation. To determine $TH_{value2}$, it is necessary to calculate the between-class variance for all amplitude values at zero crossing point from second-order differential coefficients. Similar to determining $TH_{value1}$, we can calculate the value at which between-class variance is maximized. The optimal threshold value for the edge detection algorithm is calculated using Otsu’s binarization method, as shown in Figure 5.

#### 3.3.3. One-Bit Error Correction

The strength of the received signal electrostatically coupled by the human body is not constant and varies depending on the surrounding environment and noise conditions. Due to this, when the signal strength of some bits decreases during communication, there is a case where valid edge information is not detected because signal strength does not satisfy the threshold value in the edge detection Algorithm 1. In this case, a communication error occurs immediately. We suggest a novel recovery method when such a communication error occurs.

**Figure 6:**
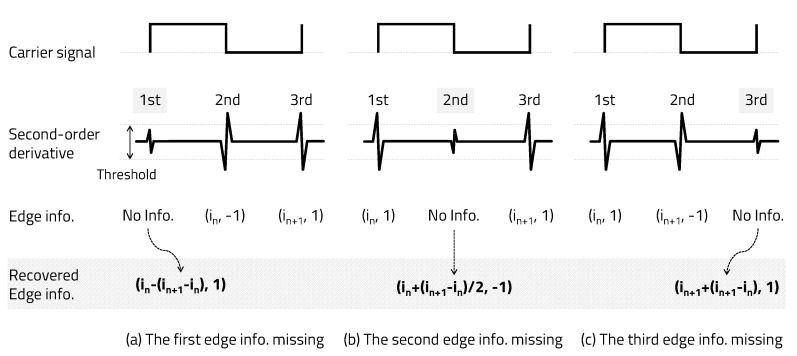
Recovery one edge information missing.

Since the carrier signal is a square wave of 50% duty ratio, 1 bit consists of three pieces of edge information, as shown in Figure 6. Let us take a look at the case where one edge information in a single bit that is the basis of the edge information recovery algorithm is missing. When only one piece of edge information among the first, second, and third edges in a single bit is missing, edge information can be recovered by utilizing a square wave with a 50% duty ratio of the carrier signal.

As shown in Figure 6a, if the first edge information is missing, the index can be recovered by subtracting the delta between the second edge index and the third edge index from the index of the second edge and can be recovered as a positive edge (1). As shown in Figure 6b, if the second edge information is missing, the index can be recovered by adding half of the delta between the first edge index and the third edge index to the index of the first edge and can be recovered as a negative edge (−1). However, assuming that the frequencies of bits 1 and 0 are integer multiples, there are two cases of second-edge information, such as a positive edge or a negative edge; thus, the edge information cannot be properly recovered. Therefore, the carrier frequencies of bits 1 and 0 should not be integer multiples of each other. Carrierfrequencyofbit1≠N×Carrierfrequencyofbit0, Carrierfrequencyofbit0≠N×Carrierfrequencyofbit1(N=integer). As shown in Figure 3, we set bit 1 to a carrier frequency of 50 kHz and bit 0 to 75 kHz. Finally, as shown in Figure 6c, if the third edge information is missing, the index can be recovered by adding the delta between the first edge index and the second edge index to the index of the second edge and can be recovered as a positive edge (1).

**Figure 7:**
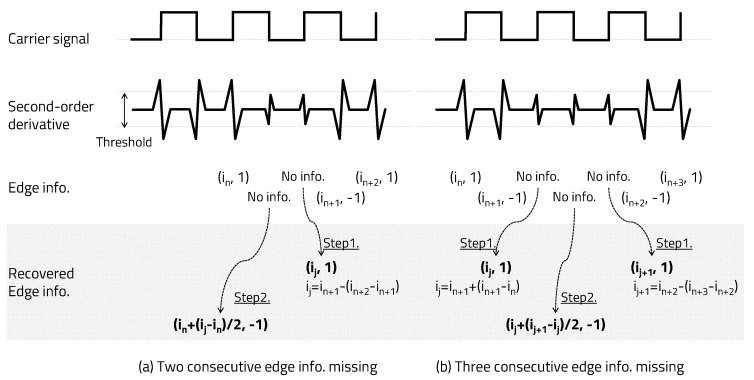
Recovery two or three edge information missing.

Only the index of a positive edge is used to determine the carrier frequency in the receiver, but the negative edge index is an important factor in recovering the missing positive edge information. When two or three pieces of edge information are missing in one bit, they can be recovered by using the edge information of the previous and next bits. As shown in Figure 7a, when two pieces of edge information are missing, they can be recovered using the second edge recovery algorithm (Step 2) after applying the first edge recovery algorithm (Step 1) described above. As shown in Figure 7b, if all three pieces of edge information points are missing, they can be recovered using the second edge recovery algorithm (Step 2) after performing the third and first edge recovery algorithms (Step 1) described above. Through the method proposed in this paper, if the error is not a continuous bit, even if up to three pieces of edge information in a bit are missing, edge information can be recovered. It is possible to recover by repeating the algorithm when one edge information in a bit is missing twice. However, if two consecutive negative pieces of edge information are missing (a total of three, including the intermediate positive edge)— that is, in the case of two or more consecutive bit errors—the method proposed in this paper cannot be corrected.

For error correction, the edge information of the previous and next bits is required. Since only one adjacent bit exists in the first and last bits of the data to be transmitted, start and stop bits are added for error correction of the first and last bits. When the carrier frequencies used for data bits 1 and 0 are used for the start and stop bits, it is impossible to distinguish between the start and stop bit and the data bit when recovering the missing edge information. Therefore, the start and stop bits have a frequency different from the carrier frequency of the valid binary data bits. Based on this, the transmission packet is composed of a total of 10 bits in the order of one start bit, eight data bits, and one stop bit.

The entire missing edge information recovery process consists of two parts. The first step is to search for edge information stored in the linked list, then find and recover a case where two or three edge information missing in a single bit. The second step is to recover a case where one edge information missing in a single bit. This recovery process allows the system to recover all missing edge information except in the case of two or more consecutive bit errors.

#### 3.3.4. Received Data Judgment

The method by which the received binary data are judged after error correction is as follows. The period of the carrier signal is calculated as the index delta between consecutive positive edges stored in the linked list. Binary data judgment is performed by comparing the period obtained through edge information stored in the linked list with the period calculated through this Equation (3). Since the receiver knows the type of carrier frequency, it is important to check that the period of the carrier frequency calculated from the edge information stored in the linked list is valid.

**Figure 8:**
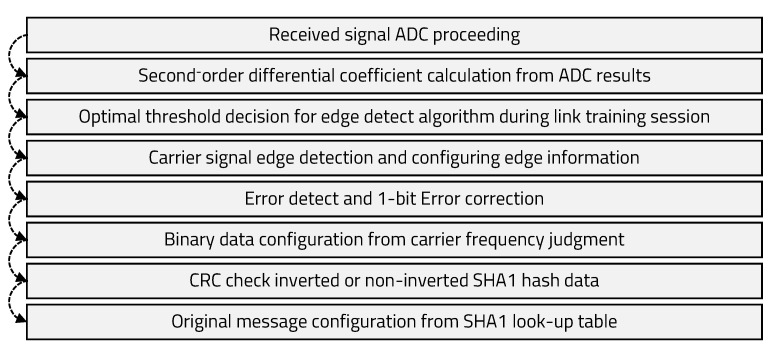
Receiver processing flow.

The algorithm for calculating and judging the period of the carrier signal from the edge information is shown in Algorithm 2. After dividing the received binary data by the CRC polynomial, it is necessary to check whether the remainder is zero. If it is not zero, the inverted SHA1 hash data should be divided by the CRC polynomial to check whether the remainder is zero. If it is zero, the original message has been successfully transmitted, and, if it is not zero, communication has failed. The entire signal processing algorithm of the receiver side is shown in Figure 8.

(3) $$Index\_delta\;=\;\frac{Rx\;ADC\;sampling\;rate}{Tx\;Carrier\;frequency}$$

**Algorithm 2:** Binary data judgment algorithm.
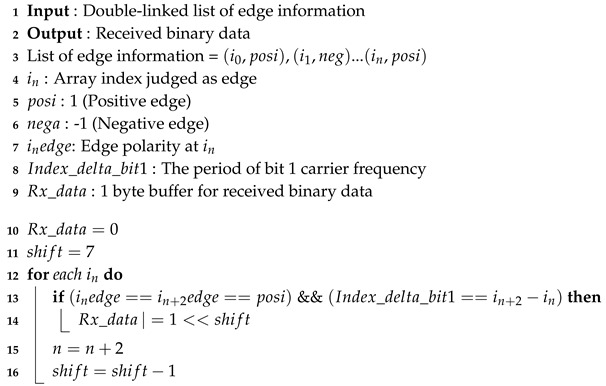


## 4. Experiment

The transmitter and receiver used STMicro’s STM32F407 board, which was designed based on the Cortex-M4 architecture. In the transmitter, the carrier frequency was set to a square wave of 50 kHz for bit 1, 75 kHz for bit 0, and 60 kHz for start and stop bits. The ADC configurations of the receiver were set to 10-bit resolution and 1.7 MSPS. Since the reference voltage of the ADC block in the MCU board is 3 V, it had a resolution of 2.93 mV per 1 ADC code, and the time interval of ADC values stored in the array through direct memory access after ADC completion was 0.595 μs. The electrode pad in contact with the human body was applied using aluminum tape. In the electrostatically coupled IBC, signal strength is affected by the current return path and the ground state of the transmitter and receiver, so the boards were powered by batteries to verify performance similar to the use environment. In addition, the onnection between the receiver board and the laptop was built using a UART-Bluetooth module (921600 BAUD) so that the verification system’s ground could also become floating free space.

**Figure 9:**
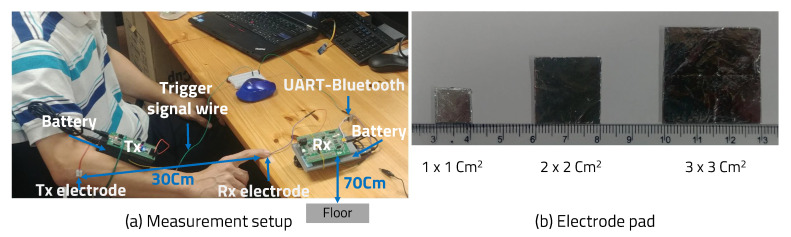
Measurement setup.

We did an experiment by attaching the Tx pad to the arm and touching the Rx pad with the finger. The receiver board was placed on a 70-cm height wooden desk, which was not a static-free desk and had no conductor properties. Only one signal was wired between transmitter and receiver and the ground was not connected to each other. The receiver used this signal to start ADC. Figure 9 shows test environment and electrode pad size.

**Figure 10:**
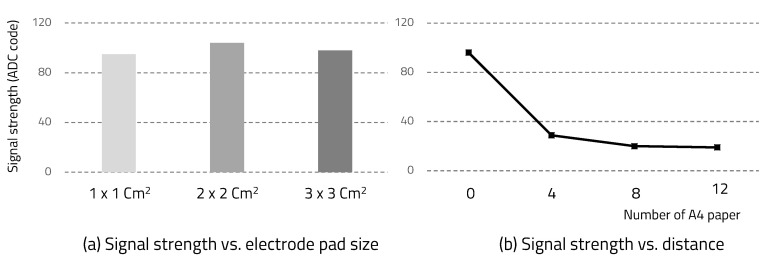
Signal strength.

Figure 10a shows the signal strength according to the electrode pad size. Using an oscilloscope to check the signal strength was considered unsuitable for application because it was well-grounded. Since the receiver performs ADC on the received signal, we were able to check the signal strength through these ADC values. Figure 10b shows the signal strength according to the distance between the skin and the electrode pad. We used A4 paper with a relative dielectric constant of around 3.0 to make the distance change between skin and electrode pad. Similar to Kurt Partridge’s study, if skin contacts the electrode pad well, the size of the pad is not a major factor, but the distance has a significant effect on signal attenuation.

**Figure 11:**
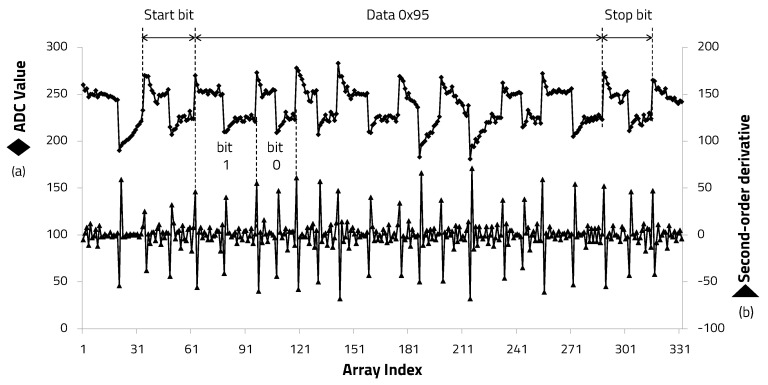
Second-order derivative from ADC results.

The demodulation process was performed by extracting edge information through the edge detection algorithm proposed in this paper from the ADC values stored in the array. After transmitting the 8-bit data hexadecimal 0 × 95, the ADC value and second-order differential coefficients of the signal transmitted to the receiver through the human body were extracted, as shown in Figure 11. The second-order derivative clearly shows the edge timing information of the square wave carrier signal. Thus, it was confirmed that it can be used for the carrier frequency determination.

For securing personal electronic authentication verification, the transmitter transmitted a total of 22 bytes of data from the ‘seongho cho’ string encrypted with SHA1 in hexadecimal “8111749a79d59f916cc7cdb4167454c271481d52” and 2 bytes of CRC-16-CCITT value in hexadecimal, “8a09”. We applied the polynomial { $x^{16}\;+\;x^{12}\;+\;x^{5}\;+\;1$ } for CRC-16-CCIT value. Then, the receiver demodulated the received signal and checked that the CRC check result was zero. If it was not zero, we re-checked that the CRC check result was zero with inverted SHA1 hash data. This personal secure electronic authentication was successfully authenticated through the proposed IBC system.

To verify the effectiveness of the error correction technique proposed in this paper, we checked the error rate after configuring a scenario wherein data were received when a person touches the electrode pad of a transmitter that sends random 8-bit binary data. After placing the receiver electrode in the pants pocket, 256 bytes of binary data were transmitted from hexadecimal 0 × 00 to 0 × FF through the human body, and the received data were output through a laptop for verification. The reason for placing the receiver electrode in the pants pocket is that it is reasonable as a way to check the effect of 1-bit error correction because the signal strength is reduced compared to when the electrode is in direct contact with the skin.

**Figure 12:**
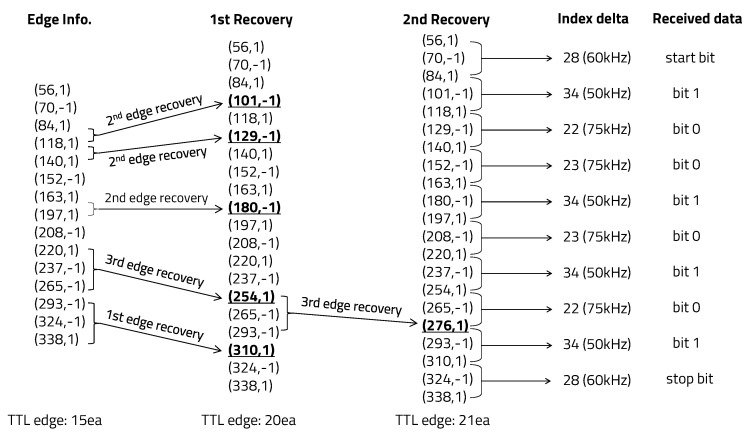
Edge information recovery of hexadecimal 0 × 95 data.

Since bit 1 was set to a carrier frequency of 50 kHz, bit 0 was set to 75 kHz, and the start and stop bits were set to 60 kHz, the index delta between consecutive positive edges determined by Equation (3) were bit 1, 34; bit 0, 22 or 23; and start and stop bits, 28 or 29. For edge detection of the received signal, the optimal threshold of the first-stage high-pass filter was set to 23 ($TH_{value1}$). The second-stage threshold was set to 48 ($TH_{value2}$), which was determined during the link training session. We repeatedly transmitted and received hexadecimal 0 × 95 binary data to verify whether any errors that occurred during communication were corrected. After connecting the laptop and the receiver board through the UART-Bluetooth module, we checked the edge information stored in the linked list. We confirmed that edge information was corrected normally by performing the edge recovery algorithm twice for non-continuous 1-bit errors, as shown in Figure 12.

To compare the error rates with and without 1-bit error correction, 256 bytes of binary data from hexadecimal 0 × 00 to 0 × FF were repeatedly transmitted 10 times and the error rates were compared. When 1-bit error correction was not applied, an average byte unit error rate of 3.5% occurred, but, when applying the 1-bit error correction algorithm, the received data error did not occur. The performance comparison with previous works is summarized in Table 1. The proposed structure does not require additional circuitry, and 1-bit error correction contributes to reliable communication.

## 5. Discussion

If an error with more than two consecutive bits occurs during 1-byte transmission, error correction is not possible, resulting in a communication error in the proposed IBC system. In this case, since the transmitter does not know whether a communication error has occurred, retransmission is not possible, and thus personal electronic authentication will fail. Therefore, there is a limitation directly applying the proposed IBC system to communication between wearable devices. To overcome these limitations, additional supplements are required for applications such as the automatic repeat request (ARQ) method, which notifies the transmitter when an error occurs at the receiver and retransmits the error block from the transmitter.

We consider it meaningful to apply the edge detection algorithm and Otsu’s binarization techniques used in computer vision to signal processing. One trigger signal was used for transmitter and receiver synchronization due to the currently implemented software constraints, therefore more studies are necessary after removing the trigger signal by implementing a continuous ADC using a circular buffer in the future. It makes it easy to apply the proposed system to the actual use environment. Even if the received signal changes after removing the trigger signal, there is no change in the carrier frequency of the received signal. Therefore, it is appropriate to verify the software-based FSK modulation, edge detection algorithm, and error correction method proposed in this paper.

## 6. Conclusions

Through this study, we implemented and verified secure electronic authentication using software-based IBC system. Personal identification data are protected by SHA1-CRC based on a random data inversion method. FSK modulation is performed using GPIO in the transmitter, and demodulation is performed using an ADC in the receiver. In addition, by applying a 1-bit error correction technique, we were able to overcome some of the external environmental conditions and noise effects and reduce the error rate of the received binary data compared to not applying error correction. When 1-bit error correction is applied, the byte error rate on the receiver is improved around 3.5% compared to not applying it. Additionally, we validated an adaptive threshold technique in which the threshold value of the edge detection algorithm is dynamically adjusted according to the strength of the received signal. It is possible to optimize demodulation performance in the receiver in various usage environments. We found that the electrode pad size does not affect the signal strength, but the distance between the electrode pad and the skin has a significant effect on the signal strength. 

## Figures and Tables

**Table 1:** Performance comparison with others.

	Zimmerman [12]	Hachisuka [20]	This Work
Communication Method	Narrowband Modulation	Narrowband Modulation	Narrowband Modulation
Modulation	OOK/DSSS	FM/FSK	FSK with software-based
Carrier Frequency	330 kHz sine wave	10.7MHz sine wave	50–75 kHz square wave
Data Rate	2.4 kbps	9.6 kbps	14 kbps
Supply voltage	9 V	3 V	5 V
Power consumption	400 mW	Not reported	480 mW
Hardware Structure	MCU and Tx/Rx circuitry	FM IC	GPIO and ADC in MCU
Error Correction	No	No	1-bit

## Abbreviations

The following abbreviations are used in this manuscript:

RF	Radio Frequency
MCU	Micro Controller Unit
FSK	Frequency Shift Keying
SHA1	Secure Hash Algorithm 1
GPIO	Gneral Purpose Input Output
CRC	Cyclic Redundancy Check
DMA	Direct Memory Acess
UART	Universal Asynchronous Receiver Transmitter
ARQ	Automatic Repeat reQuest
